# Chest X-ray Classification for the Detection of COVID-19 Using Deep Learning Techniques

**DOI:** 10.3390/s22031211

**Published:** 2022-02-05

**Authors:** Ejaz Khan, Muhammad Zia Ur Rehman, Fawad Ahmed, Faisal Abdulaziz Alfouzan, Nouf M. Alzahrani, Jawad Ahmad

**Affiliations:** 1 School of Engineering, RMIT University, Melbourne 3000, Australia; s3738707@student.rmit.edu.au; 2 Department of Electrical Engineering, HITEC University, Taxila 47080, Pakistan; mzia.rehman@hitecuni.edu.pk; 3 Department of Cyber Security, Pakistan Navy Engineering College, NUST, Karachi 75350, Pakistan; fawad@pnec.nust.edu.pk; 4 Department of Forensic Sciences, College of Criminal Justice, Naif Arab University for Security Sciences (NAUSS), Riyadh 14812, Saudi Arabia; falfouzan@nauss.edu.sa; 5 Information Technology Department, Collage of Computer Science and Information Technology, Al Baha University, Al Bahah 65731, Saudi Arabia; noufalzahrani@bu.edu.sa; 6 School of Computing, Edinburgh Napier University, Edinburgh EH10 5DT, UK

**Keywords:** COVID-19, deep learning, classification, transfer learning, chest X-rays

## Abstract

Recent technological developments pave the path for deep learning-based techniques to be used in almost every domain of life. The precision of deep learning techniques make it possible for these to be used in the medical field for the classification and detection of various diseases. Recently, the coronavirus (COVID-19) pandemic has put a lot of pressure on the health system all around the world. The diagnosis of COVID-19 is possible by PCR testing and medical imagining. Since COVID-19 is highly contagious, diagnosis using chest X-ray is considered safe in various situations. In this study, a deep learning-based technique is proposed to classify COVID-19 infection from other non-COVID-19 infections. To classify COVID-19, three different pre-trained models named EfficientNetB1, NasNetMobile and MobileNetV2 are used. The augmented dataset is used for training deep learning models while two different training strategies have been used for classification. In this study, not only are the deep learning model fine-tuned but also the hyperparameters are fine-tuned, which significantly improves the performance of the fine-tuned deep learning models. Moreover, the classification head is regularized to improve the performance. For the evaluation of the proposed techniques, several performance parameters are used to gauge the performance. EfficientNetB1 with regularized classification head outperforms the other models. The proposed technique successfully classifies four classes that include COVID-19, viral pneumonia, lung opacity, and normal, with an accuracy of 96.13%. The proposed technique shows superiority in terms of accuracy when compared with recent techniques present in the literature.

## 1. Introduction

COVID-19 is the most recent viral outbreak, and started in the city of Wuhan, China. Within the span of a few months, it spread to almost all parts of the world [1]. It is a highly contagious disease and transmits by exhaling droplets while respiring. The sudden spike of this highly contagious infection created a global health crisis [2]. According to recorded data, COVID-19 caused over 0.3 million deaths globally within two months after being recognized globally as a pandemic by the World Health Organization (WHO) [3,4]. This infection is commonly known as COVID-19, its scientific name is severe acute respiratory syndrome coronavirus 2 (SARS-CoV-2) and it belongs to the coronaviridae family [2]. By 8 December 2021, it had infected over 267 million people around the globe and caused over 5.2 million deaths [3]. COVID-19 negatively affects every sector of life around the globe due to restrictions enforced by governments that are aimed at avoiding exposure of people to this contagious infection [5]. Illnesses that can be classified as being caused by COVID-19 can vary from mild to severe and critical illnesses. COVID-19 can cause pneumonia. The name pneumonia originates from the Greek word pneumon, which means the lungs. Therefore, pneumonia is related to lung disease. Pneumonia causes inflammation in the lungs, which hinders respiration [6]. Exposure to chemicals and food aspirations are other causes of pneumonia. As mentioned earlier, pneumonia causes inflammation in the lungs, which leads to the lungs’ alveoli being filled with fluid or sticky substance (i.e., pus). The sticky fluid causes hindering in the exchange of carbon dioxide and oxygen between the blood and lungs. Reduced exchange of carbon dioxide and oxygen hampers the ability to breathe [7].

Different pathogens, like bacteria, fungi and viruses, cause pneumonia and each of these pneumonias are treated differently. To treat bacterial pneumonia, antibiotics are used. Antifungal drugs are used to treat pneumonia that is caused by fungi, while antiviral drugs are used to treat viral pneumonia [8]. For the diagnosis of pneumonia, a number of techniques have been adopted, including CT scan, chest X-rays, sputum test, complete blood picture, blood gas analysis, and others. However, for the detection of COVID-19, reverse transcription-polymerase chain reaction (RT-PCR) testing is considered reliable, although it is not 100% accurate. A RT-PCR test is used to detect genetic information regarding SARS-CoV-2 from the upper respiratory tract [9].

There is a need to develop a technique that can help medical staff in the diagnosis of COVID-19. Early diagnosis of COVID-19 can save a patient’s life by providing on-time necessary medical attention. Recently, deep learning has emerged as one of the techniques to be used for image processing tasks. It has been found that it produces significant outcomes in different fields, including agriculture [10], medicine [11,12], gesture recognition [13], and remote sensing [14], etc. In the medical field, it is used for the detection and classification of different diseases, including skin diseases [15,16], different types of ulcers, and cancer [17], etc. These deep learning techniques significantly help doctors to diagnose diseases efficiently. Human errors can also be avoided by using deep learning techniques for the detection of different diseases. As mentioned earlier, CT scan and chest X-rays can be used to detect pneumonia. Pneumonia caused by COVID-19 is intense and affects the lungs significantly very quickly. The major difference between typical pneumonia and pneumonia caused by COVID-19 is that pneumonia caused by COVID-19 affects the whole lungs while typical pneumonia damages only part of the lungs.

## 2. Related Work

Mahmud et al. [18] proposed a deep learning-based technique for the classification of COVID-19 and pneumonia infection. Features are extracted using a deep CNN model named CovXNet. A public dataset is utilized for training containing 1493 samples of non-COVID-19 pneumonia and 305 samples of COVID-19 pneumonia. The model successfully classified non-COVID-19 pneumonia and COVID-19 pneumonia with an accuracy of 96.9%. Umair et al. [19] presented a technique for the binary classification of COVID-19. A publicly available dataset is used for training and evaluation of the technique, consisting of 7232 chest X-ray images. Four deep learning models are being compared in this study. Various evaluation parameters are utilized for the validation of results. Li et al. [20] proposed a technique for the detection of COVID-19 infection. The proposed technique successfully differentiates COVID-19 pneumonia and community-acquired pneumonia (CAP). The deep learning model that is utilized for training is named COVNet; this is three-dimensional CNN architecture. A publicly available dataset is used which contains CT scan samples of COVID-19 and community acquired pneumonia (CAP). The COVNet model attained a rate of 90% sensitivity and 96% specificity.

Abbas [21] proposes another convolution neural network-based technique for the classification of COVID-19 infection using chest X-ray images. The CNN model named decompose, transfer, and compose, and commonly known as DeTrac, was used. Multiple datasets from various hospitals throughout the world were used in this research. The DeTrac model attained an accuracy of 93.1% and a sensitivity of 100%. To classify COVID-19 and typical pneumonia, Wang et al. [22] presented a technique based on deep learning which used the inception model [23]. Modifications in fully connected layers of inception are completed before training the network. In this study, 1053 images were used. The model gave an accuracy of 73.1% with a sensitivity of 74% and specificity of 67%. Sankar et al. [24] proposed a deep learning technique for the classification of COVID-19 infected chest X-rays. A Gaussian filter was used for preprocessing, while the local binary pattern was utilized to extract texture features. Later, the extracted LBP features are fused with the CNN model InceptionV3 to improve the performance. The classification is carried out using multi-layer perceptron. The model was validated on an X-ray dataset and attained an accuracy of 94.08%. Panwar [25] proposes a convolutional neural network-based technique where a 24 layer CNN model has been used for the classification of COVID-19 and normal images. The author named this model nCOVnet. The X-ray dataset was used for training nCOVnet. The model attains an accuracy of up to 97%. Zheng [26] presents the segmentation-based classification technique. The U-Net [27] is trained on CT images to generate lung masks. Two-dimensional U-Net is used for this purpose. Later, the mask generated by U-Net is fed to DeCoVNet for the classification of COVID-19. The architecture of DeCoVNet consists of three parts: (1) the stem network, consisting of 3-D vanilla, along with a batch norm and pooling layer; (2) two 3D ResBolcks are used in the second stage, where ResBolcks are used for feature map generation; (3) the third part of DeCoVNet is used for classification that is based on probabilities. A progressive classifier is used for the binary classification of COVID-19.

Xu et al. [28] proposed a technique for the detection of COVID-19 infection using the deep learning-based model. Two ResNet [29] based models were used in this study: (1) ResNet18; (2) a modified ResNet18 with the mechanism of localization. The CT scan images were used for training the models. The final evaluation is performed using noisy-OR Bayesian. The overall accuracy of the proposed technique is cited as 88.7%. Hussain et al. [30] proposed a system that is called CoroDet and is based on convolutional neural networks for the detection of COVID-19 infection. The proposed CNN model is comprised of 22 layers, and is trained on chest X-rays and CT scan images. The model is able to classify COVID-19 and non-COVID-19, Moreover, it can classify three different classes, including COVID-19, pneumonia, and normal. The 22 layered model shows good classification results. Khan et al. [31] presented a technique for the classification of COVID-19 disease. The proposed technique used CNN for the classification; a known deep learning model Xception is modified for this purpose. The modified model is named CoroNet by the authors. The dataset used for training consists of four classes, including COVID-19, normal, viral pneumonia, and fungal pneumonia. Using the mentioned dataset, the model is trained using the different combinations of datasets. The model gave 89.6% accuracy. Choudary et al. [32] adopted a deep learning technique to classify COVID-19 and viral pneumonia. Various deep learning models have been used for training in this work. In addition, the transfer learning approach is exploited for training deep learning models. The public dataset is utilized for the training of models. The dataset contains samples of COVID-19, typical viral pneumonia, and chest X-rays of healthy and normal people. The models attained good classification accuracies. Ozturk et al. [33] presented a 17 layered Darknet model for the detection of COVID-19 infection. Different sizes of filters were employed at CNN layers. The presented technique classifies binary classes (COVID-19 and no finding) and multiple classes (COVID-19, pneumonia, and no findings). For model training, raw chest X-ray images were used. The model attained an accuracy of 98.08% for binary classification, while for multiple classes an accuracy of 87.02% is achieved.

For detection of COVID-19, the majority of research has been performed using chest X-rays, which show the importance of chest X-rays in diagnosing chest infections and, specifically, for diagnosing COVID-19. The chest X-rays were found to be the primary tool in medical image analysis. Traditional image processing-based feature extraction techniques are complex compared to deep learning techniques. Recently deep learning techniques surpass traditional techniques in terms of performance. However, traditional techniques can be used, along with deep learning techniques, for aid [24]. Moreover, deep learning techniques require a large amount of data for training and testing. Deep learning models trained on the limited datasets are not generalized; thus, such models are not reliable. It has been found through the literature, that data augmentation techniques can be used to resolve small dataset issues [34]. Furthermore, the already available research is more focused on the binary classification of COVID-19 [18,19,20,21,22] and limited research has been conducted for multiclass classification of COVID-19 [28,29,30,31,32,33]. The performance of multiclass classification is not yet adequate, and hence their performance needs to be improved.

## 3. Materials and Methods

In this section, the dataset used for the training and testing are discussed along with the deep learning models used in this study. The dataset used in this study acquired from Kaggle is composed of multiple datasets. The dataset is further discussed in Section 3.1. Likewise, the proposed methodology for the classification of COVID-19 infection is discussed in Section 3.2.

### 3.1. Dataset

The dataset used for training and evaluation of the proposed technique is publicly available on Kaggle [32,35]. This dataset has been revised thrice; the dataset used in this work is acquired from Kaggle after recent revisions. The mentioned dataset is composed of multiple sub-datasets, with four different classes, including COVID-19, lung opacity, normal and viral pneumonia. It is important to discuss the composition of the used dataset in detail as it is made by merging different datasets. Each class is created by merging different sub-datasets. The class COVID-19 contains a total of 3616 images, which are gathered from four different sources. The BIMCV-COVID19+ [36] dataset largely contributes to the used COVID-19 dataset with 2473 images. It is one of the largest independent datasets that is publicly available. Other datasets, which contribute to this COVID-19 dataset, are the German Medical School dataset [37] with 183 chest X-ray images, while 560 chest X-ray images are gathered from SIRM, GitHub, Kaggle, and Twitter [38,39,40,41]. In addition, another dataset is available on GitHub [42] containing 400 chest X-ray images which have been merged.

**Figure 1:**
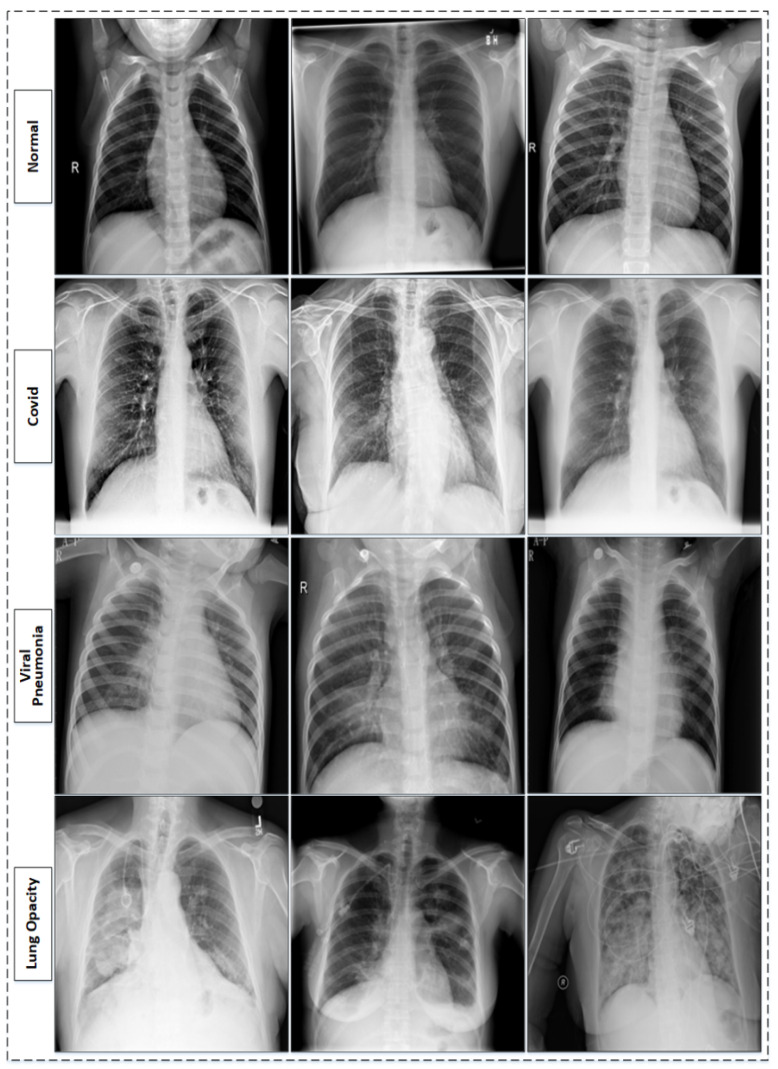
Sample images of original dataset [32,35].

The RSNA pneumonia challenge dataset is one of the known chest X-ray datasets [43]. The RSNA dataset consists of different lung abnormalities and normal lungs (healthy lungs). The abnormalities range from different lung infections to lung cancer. It contains 26,684 chest X-ray images in the Dicom format; further, the dataset is divided into three major categories. The largest of these categories contains 11,821 images with different lung infections and, of these, 6012 images are categorized as non-COVID-19 lung infection (lung opacity), while 8851 images are normal and healthy lungs. The dataset is examined by medical experts based on key symptoms, and clinical history is also considered during inspection, as it is important to know whether or not the patient has ever suffered from any correlated infections before. The dataset is further extended by adding 1341 normal chest X-ray images, along with viral pneumonia chest X-ray images, which are 1345 total in number and are sourced from [44]. The sample images from the used dataset [35] are shown in Figure 1. The composition of the dataset [35] is also presented in Table 1.

### 3.2. Proposed Methodolgy

**Figure 2:**
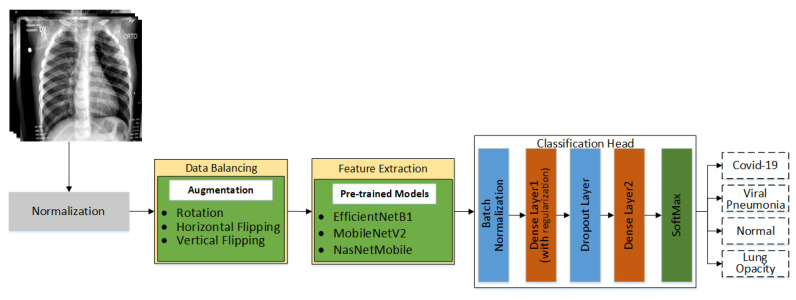
Detailed workflow of proposed technique.

In this proposed work, a multiclass classification technique for chest X-rays is proposed. The main goal is to identify X-rays infected by COVID-19. In the literature, a significant amount of research related to the binary classification of COVID-19 is present, but it is still difficult to find research that is related to the multiclass classification of COVID-19. Multiclass classification of X-rays is a challenging task as there are inter-class similarities. In addition, the availability of datasets is one of the major problems. The datasets publicly available are highly class imbalanced; this is one of the other challenges to deal with while working on multiclass classification. Different pre-trained models are being used in this work to evaluate their performance on chest X-ray for the classification of chest infections, including COVID-19. The proposed workflow of this research is shown in Figure 2. The details of every step shown in Figure 2 are discussed in the following subsections.

#### 3.2.1. Data Normalization and Augmentation

Acquiring datasets for training deep learning models is not easy, as datasets are not always readily available. The deep learning models require quality datasets with a large number of samples for efficient training [10]. The dataset is normalized within a range of 0 and 1. Every pixel of images present in the dataset are multiplied by a factor of 1/255. This has been done to make the dataset consistent in terms of pixel intensity. The acquired dataset is a class imbalance; such a dataset contains the different numbers of images in each class. The deep learning models cannot be efficiently trained on such datasets, as they are biased toward one or more classes, which significantly affects the performance of the model. Moreover, deep learning models require a significant amount of a dataset for training; otherwise, overfitting can play a role in deteriorating the performance of the model.

To overcome both of these above-mentioned problems, the image augmentation approached is adopted. Image augmentation not only increases the amount of the dataset but also helps in making the dataset class balanced. The image augmentation approach enables the addition of more examples to the classes, which originally have fewer examples, thus enhancing the quality and size of the original dataset, which significantly affects the performance of the model. There are a number of image augmentation techniques. Image augmentation techniques are used according to requirements. In this work, the image dataset is used for training. Choosing a good image augmentation technique is critical; otherwise, it can impact the model performance negatively. A good augmentation technique is meant to preserve all of the information that is present in the original data, however, it also increases the size of the dataset.

**Figure 3:**
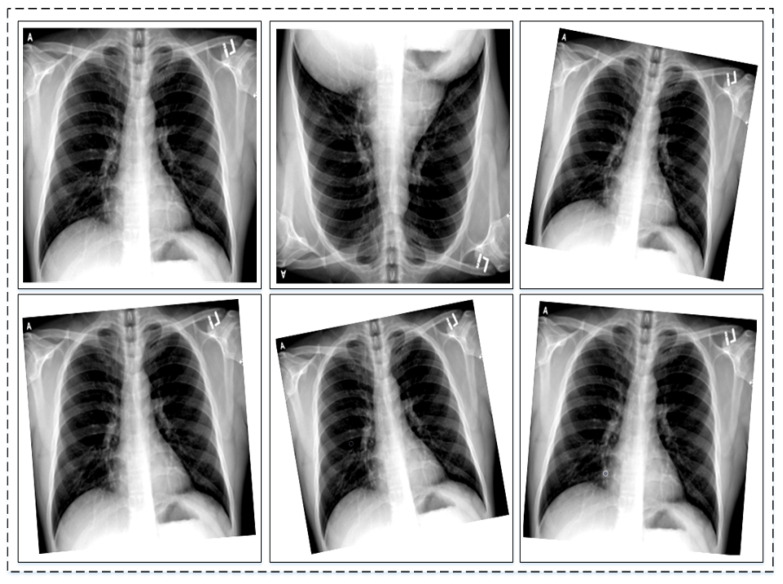
Sample images of original dataset after performing data augmentation.

To address the class imbalance challenge, data augmentation is applied to three classes, which are the COVID-19 class, viral pneumonia, and lung opacity. Augmentation carried out on images is known as image augmentation and it has various types. The types include geometric or positional augmentation, random erasing, color space transformation, mixing images, and kernel filter-based augmentation [45]. Geometric augmentation has been found suitable for datasets that are similar to the one used in this work. Geometric augmentation also has various types, including rotational, translation, scaling, cropping, and flipping. Depending upon the need and nature of the original dataset these types can be used. As in this work, the original dataset has inter-class similarities. Keeping this in mind, only two geometric augmentations have been found to be useful; these are rotational and flipping augmentation. In rotational augmentation, images can be rotated clockwise and anti-clockwise with different degrees of angle. Practically, it is possible to rotate images from 1 to 359 degrees, but to preserve augmentation safety, rotation within 20 degrees is suitable. On the other hand, flipping can be horizontal and vertical around the axis. It has been found to be useful when applied on datasets, like Cifar [46] and ImageNet [47]. Flipping is label preserving except for text, but it is important to check the augmentation manually. The nature of the dataset is important when applying augmentation to preserve labels of the dataset. Generally, geometric augmentation is suitable and effective where position bias is present in the dataset. A few examples of augmentation are shown in Figure 3.

#### 3.2.2. Feature Extraction and Classification of Chest X-rays

Deep learning models extract features from the images using convolutional layers; based on these features these model also classify images. The initial layers of DL models extract edges, contours, etc., while later layers extract more detailed attributes of images. In this study, three different deep learning models have been used for the feature extraction and classification of chest X-rays. The models used are EfficientNetB1 [48], NasNetMobile [49], and MobileNetV2 [50]. The architecture of the used models are discussed briefly. The MobileNetV2 is a lightweight model, consisting of 17 bottlenecks. These bottlenecks are made of pointwise and depth-wise convolution layers along with Relu6 and batch normalization layers. The model consists of a total of 53 layers. On the other hand, NasNetMobile is made up of two different cells, called normal and reduction cells. The reduction cells are followed by four normal cells throughout the architecture. The reduction cells reduce the dimension of the feature map by a factor of 2. The third model used in this work is EfficientNetB1, which is a variant of the EfficientNetB0 baseline. The MBConv block is the core of EfficientNetB1, an inverted residual block used to reduce the number of trainable parameters. The squeeze and excitation block is the part of the MBConv block which aids in feature extraction by giving weights to channels of the MBConv block. The EfficientNetB0 baseline uses compound scaling, which is a combination of width, depth and resolution scaling. In EfficientNetB1, the swish activation function is used, which is the combination of linear and sigmoid activation functions. The swish activation function helps retain the negative values. EfficientNetB1 uses an input size of 240 × 240. The architecture of the EfficientNetB0 baseline is presented in Table 2.

**Figure 4:**
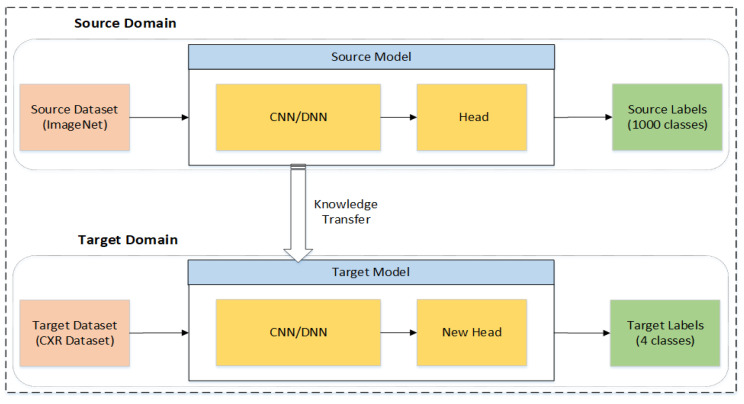
Illustration of transfer learning.

These models are previously trained on the large dataset ImageNet [38]. All three models have been retrained using the concept of Transfer Learning (TL). TL helps the deep learning model obtain better outcomes by reusing the deep learning model’s previously gained information on huge datasets [51]. The TL technique not only improves results but also reduces the training time significantly. The concept of TL is illustrated in Figure 4, where there are two domains: the source domain and the target domain. The source domain transfers the knowledge to the target domain. Both domains have three parts, including the model, dataset, and labels [52]. The models used in the source domain are trained from scratch on a large dataset, where the labels are the categories of that dataset, as in the case of the ImageNet dataset which has 1000 categories. The models are retrained after performing augmentation on the original dataset.

Later, the models are fine-tuned according to needs of this study. The classifier head is removed from all of the three networks, which consists of fully connected layers and global average pooling layers. Two new fully connected layers are added named the Dense layer1 and Dense layer2 of size 256 and 4, respectively. The last fully connected layer is activated using the softmax function, while the Adam optimizer is used in this work. The batch normalization layer is employed before fully connecting the layers in order to normalize the output of previous layers. Introduction of the batch normalization layer not only reduces the convergence time but also improves the accuracy. The dropout layer is employed after the first fully connected layer to make the model generalized and to avoid overfitting. The cross entropy function is used as a cost function. It is represented mathematically in the following equation:

(1) $$H\left(P,Q\right)=-\sum_{c}^{N}P\left(o,l\right)\log Q\left(o,l\right),$$

where *N* represents total classes, the labels of classes are denoted by *l*, *P*(*o*, *l*) is the true probability of observation *o* over class *c* while *Q*(*o*, *l*) is the predicted probability of observation *o* over class *c*. The hyperparameter tuning plays an important role while training the deep learning models. Learning rate is one of the important parameters, instead of using one learning rate throughout the training; a learning scheduler is used in this work. It is designed in such a way that whenever the validation loss stops reducing, the learning rate is divided by a factor of 2. The starting learning rate used in this work is 0.0001. It has been found during experimentation that small learning rates are better when using pre-trained models, as this helps to retain much of the information from the previously trained model. Higher learning rates makes the training faster but can also cause weights to explode during training, which affects the training process adversely.

#### 3.2.3. Experimental Setup

As mentioned earlier, the chest X-ray dataset with four classes is taken from Kaggle. The dataset went through the preprocessing stage, which address the problem of class imbalance. This is an important step, as an imbalanced dataset adversely affects the model training by showing bias towards one or more classes. Later, the dataset is split into three subsets—training, validation, and testing—with a ratio of 70:20:10, respectively. The testing dataset is unseen, and is used for the evaluation of the model after training the models. The experiments are performed on Intel Core I7, which has 16 gigabytes of RAM. The system is also supported by NVIDIA GTX 1070 Ti. Three different deep learning models are used to compute the results using TL. Moreover, two different training strategies have been adopted in this work. In Strategy I, all three models have been trained using TL on the preprocessed datasets (normalization and data augmentation) and classified using the classification head without batch normalization and the dropout layer, and also without the L2 regularization technique. In the Strategy II, all three models are trained again and classification is performed using a classification head with the batch normalization and dropout layer. Moreover, the Dense layer1 is also regularized using the L2 regularization technique, which is also known as weight decay. The results are evaluated using various evaluation parameters to validate the results of the proposed technique. The evaluation parameters used in this work are accuracy, precision, FPR, sensitivity, and specificity.

## 4. Results

In this section, the classification performance of the proposed technique on a multiclass chest X-ray image dataset is discussed in detail. The classification results are presented in Section 4.1 while Section 4.1.1 presents the findings of experiments along with comparisons of other techniques.

### 4.1. Classification Results

**Figure 5:**
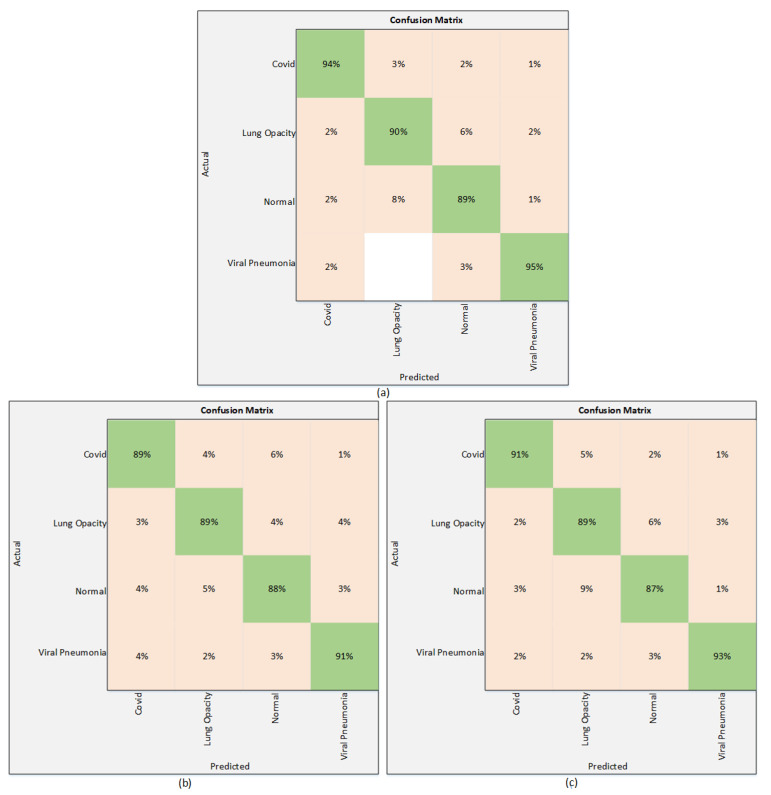
Confusion matrix using Strategy I. (**a**) EfficientNetB1 (**b**) NasNetMobile (**c**) MobileNetV2.

The results of the proposed technique are presented in this section. The results obtained using Strategy I are presented in Table 3. All three models are trained without employing any regularization technique. Table 3 shows that among these three deep learning models, EfficentNetB1 outperforms the others with the highest test accuracy of 92%. It attains a precision of 91.75%, while the sensitivity and F1 scores are 94.5%, and 92.75%, respectively. On the other hand, NasNetMobile provided an accuracy of 89.30% and a precision of 89.25%. The sensitivity is recorded as 91.75% and NasNetMobile gave an F1 score of 91%. The third model used in this work is MobilNetV2, which performs relatively better than NasNetMobile. The MobileNetV2 achieves an accuracy of 90.03%, whereas the precision is recorded as 92.25%. The sensitivity attained using MobileNetV2 is 92% and the F1 score is recorded as 91.75%. It is clearly seen in Table 3 that EfficientNetB1 shows superiority over other models in terms of accuracy, along with other parameters. These results are also validated using a confusion matrix. The confusion matrix is shown in Figure 5.

**Figure 6:**
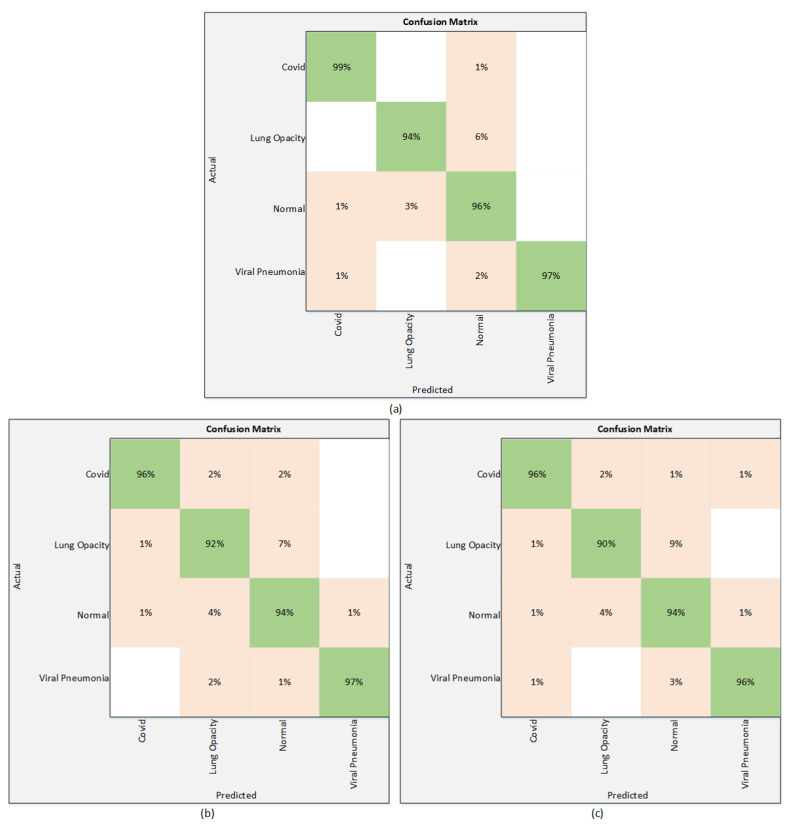
Confusion matrix using Strategy II. (**a**) EfficientNetB1 (**b**) NasNetMobile (**c**) MobileNetV2.

Results of the proposed technique using the Strategy II are presented in Table 4. As mentioned in Section 3, to improve the performance of the models used in Strategy I, the classification head is modified by employing a normalization layer along with the dropout layer. This strategy significantly improves the results of these three models. According to Table 4, EfficientNetB1 attains the highest test accuracy of 96.13%, while the precision, sensitivity, and F1 score are 97.25%, 96.50%, and 97.50%, respectively. Among the other two models, NasNetMobile performs better than MobileNetV2 and attains the accuracy of 94.81%. The value of precision is 95.5%, whereas the sensitivity and F1 score are 95% and 95.25%, respectively. The accuracy and precision attained by the MobileNetV2 are 93.96% and 94.50%, while the sensitivity and F1 score are 95% and 94.50%, respectively. The results of Table 4 can also be validated through a confusion matrix. The Figure 6 presents the confusion matrices of all three models.

**Figure 7:**
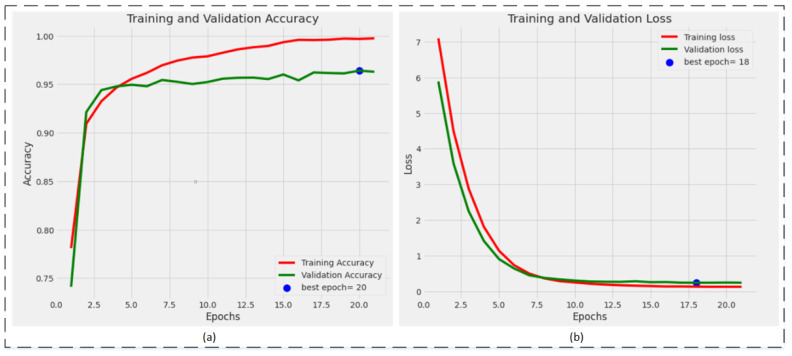
Training plots of the best performing model, EfficientNetB1: (**a**) accuracy plot; (**b**) loss plot.

The training plots of the best-performing model, EfficientNetB1, are shown in Figure 7. Figure 7a represents the training and validation accuracy plot. The validation accuracy is achieved by the model on the 20th epoch, while Figure 7b represents the training and validation loss plot. The minimum validation loss is attained on the 18th epoch, while the training continues until the 21st epoch but after the 18th epoch, the loss did not reduce further.

#### 4.1.1. Analysis and Comparison

**Figure 8:**
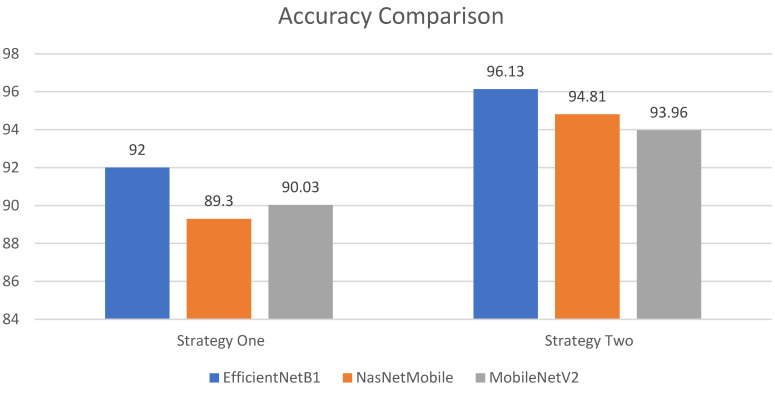
Performance comparison between Strategy I and Strategy II.

In this subsection, the observations during training are stated. In this work, three different models are trained by opting for two different strategies. It has been observed that the performance of all three models significantly improves after employing regularization techniques. Though EfficientNetB1 performs well in both scenarios, regularization techniques significantly improve the performance of EfficientNetB1. Moreover, it also has been observed that the number of the parameters in the model is not related with the performance of the model, as in Strategy I, MobileNetV2 has fewer parameter than NasNetMobile, and attains better accuracy than NasNetMobile. A comparison of the performance of the models are shown in Figure 8. It is clearly seen that a modified EfficientNetB1 outperforms in both strategies. In addition, a comparison of performance with the recent work that is presented in the literature is shown in Table 5.

## 5. Conclusions

A deep learning-based technique is proposed for the classification of different chest infections. The proposed automated system can differentiate chest infections after the evaluation of chest X-ray images. Pixel normalization is used as a preprocessing tool to normalize the pixel intensity of images as the data are gathered from different sources. Moreover, image augmentation is adopted to resolve the class imbalance problem. Three pre-trained deep learning models—EfficeintNetB1, NasNetMobile, and MobileNetV2—are fine-tuned and later retrained to perform the classification of four different chest X-ray classes. This research shows the performance of all three models is improved after applying regularizing techniques to the models. The regularized EfficientNetB1 model outperforms the other models with a classification accuracy of 96.13%, and also when compared with other techniques, the proposed technique shows its superiority in performance.

In the future, this study can be extended for a larger database with more than four classes to be classified. In addition, other lightweight deep learning models can be used to improve the computational time required. Moreover, for the improvement in performance some optimization techniques, specifically metaheuristic techniques, can be employed to select optimum features for classification.

## Figures and Tables

**Table 1:** Composition of COVID-19 dataset [32,35].

Data Composition	BIMCV-COVID19+ [36]	German Medical School [37]	SIRM, GitHub, Kaggle, and Twitter [38,39,40,41].	GitHub [42]	RSNA [43]	Kaggle [44]	Total
**COVID-19**	2473	183	560	400			3616
**Lung Opacity**					6012		6012
**Normal**					8851	1341	10,192
**Pneumonia**							1345

**Table 2:** Architecture of EfficientNetB0 Baseline.

Stage	Operator	Resolution	Channel	Layers
1	Conv3 × 3	224 × 224	32	1
2	MBConv1, k3 × 3	112 × 112	16	1
3	MBConv6, k3 × 3	112 × 112	24	2
4	MBConv6, k5 × 5	56 × 56	40	2
5	MBConv6, k3 × 3	28 × 28	80	3
6	MBConv6, k5 × 5	14 × 14	112	3
7	MBConv6, k5 × 5	14 × 14	192	4
8	MBConv6, k3 × 3	7 × 7	320	1
9	Conv1 × 1 & Pooling & FC	7 × 7	1280	1

**Table 3:** Classification results using Strategy I.

Deep Learning Models	Evaluation Parameters			
	Accuracy	Precision	Sensitivity	F1 Score
EfficientNetB1	92%	91.75%	94.50%	92.75%
NasNetMobile	89.30%	89.25%	91.75%	91%
MobileNetV2	90.03%	92.25%	92%	91.75%

**Table 4:** Classification results using Strategy II.

Deep learning Models	Evaluation Parameters			
	Accuracy	Precision	Sensitivity	F1 Score
EfficientNetB1	**96.13%**	97.25%	96.50%	97.50%
NasNetMobile	94.81%	95.50%	95%	95.25%
MobileNetV2	93.96%	94.50%	95%	94.50%

**Table 5:** Comparison with other techniques.

Reference	Year	# of Classes	Accuracy
Khan et al. [31]	2020	4	89.6%
Rahman et al. [53]	2021	3	96.29%
Abbas et al. [21]	2021	3	93.1%
**Proposed**	**2022**	**4**	**96.13%**

## Data Availability

The dataset used in this research is publicly available with name “COVID-19 Radiography Database | Kaggle” on https://www.kaggle.com/tawsifurrahman/covid19-radiography-database.
